# Defatted black soldier fly (*Hermetia illucens*) diets improved hemato-immunological responses, biochemical parameters, and antioxidant activities in *Streptococcus iniae*-infected Nile tilapia (*Oreochromis niloticus*)

**DOI:** 10.1186/s12917-025-04484-7

**Published:** 2025-02-25

**Authors:** Eman A. Abd El-Gawad, Eman Zahran, Hadeer Youssuf, Ahmed Shehab, Aya F. Matter

**Affiliations:** 1 Department of Aquatic Animal Medicine, Faculty of Veterinary Medicine, Benha University, Toukh, Kalubia Egypt; 2 Department of Aquatic Animal Medicine, Faculty of Veterinary Medicine, Mansoura University, Mansoura, 35516 Egypt; 3 Department of Nutrition and Clinical Nutrition, Faculty of Veterinary Medicine, Benha University, Toukh, Kalubia Egypt

**Keywords:** Insect meal, Immunity, Feeding, Gene expression, Bacterial challenge, Nile tilapia

## Abstract

**Background:**

Challenges of limited supply and increasing prices of fishmeal have driven the aquaculture nutritionists to seek alternative sustainable protein rich ingredients to keep manufacturing aquafeeds in a maintainable and cost-effective way. Black soldier fly, Hermetia illucenslarvae meal represent great potential as a sustainable alternative to fishmeal in aquafeeds.

**Methods:**

Three replacement diets for fishmeal were prepared at different levels of defatted black soldier fly (Hermetia illucens) meal (DBSFM): Diet 1 (0 g DBSFM /kg diet, control), 33% (DBSFM-33%, 66 g DBSFM /kg diet), and 100% (DBSFM-100%, 200 g DBSFM /kg diet) to investigate their effects on biochemical parameters, immuno-hematological responses, antioxidant activities, and inflammatory gene expression in Nile tilapia, *Oreochromis niloticus*, a total of 270 (40.0 ± 0.50 g) before and after challenge with *Streptococcus iniae (S. iniae*). The feeding trial lasted six weeks (pre-challenge) and two weeks (post-challenge).

**Results:**

The results showed a significant improvement in white blood cell count (*P* < 0.01), lymphocyte count (*P* < 0.01), serum lysozyme activity (*P* < 0.001), and phagocytic activity (*P* < 0.001), mostly in the DBSFM-100% group following the pre-challenge phase compared to the control group. Post-challenge phase exhibited significant increases in blood indices in the DBSFM-treated groups compared to the control group. Following pre- and post-challenge periods, both DBSFM-supplemented groups experienced significant increases (*P* < 0.01, *P* < 0.001), in serum total protein levels. Albumin and globulin levels also experienced similar increases (*P* < 0.05, *P* < 0.01), but only post-challenge. Total antioxidant capacity exhibited a significant increase in both DBSFM-supplemented groups following the post-challenge, as did superoxide dismutase, catalase, and glutathione peroxidase in the liver and spleen. Conversely, levels of glucose, cortisol, and malondialdehyde followed the opposite trend. DBSFM-100% inclusion revealed significant (*P* < 0.05) up-regulation of interleukin 1β *(IL-1β)* in the pre-challenge phase compared to control, but no significance (*P* > 0.05) was seen for other genes. Anti-inflammatory-related genes transforming growth factor*-β* and interleukin-10 mRNA expression levels were up-regulated in DBSFM-supplemented groups compared to the control post-challenge, but the opposite was seen for *IL-1β* and tumor necrosis factor- α.

**Conclusion:**

These findings suggest that Nile tilapia challenged with *S. iniae* may experience significant enhancements in hemato-immunological parameters, antioxidant capability, and anti-inflammatory gene expression when fish meal is replaced with DBSFM up to 100%.

## Introduction

Addressing bacterial infections is crucial in the aquaculture industry, impacting both freshwater and marine fish and resulting in substantial financial losses [1]. Streptococcosis is a severe bacterial disease caused by the *Streptococcus* genus, affecting the sustainability of freshwater and marine fish farming worldwide [2–4]. *Streptococcus iniae* (*S. iniae*) is a significant pathogen in cultured Nile tilapia and possesses zoonotic importance [5]. *S. iniae* is an ecologically versatile pathogen that can thrive in fresh and marine waters. This ecological versatility has contributed to its widespread distribution in different geographic areas and has led to repeated epizootics in aquaculture. The ability of the bacterium to infect several host species complicates control measures and enhances the potential for outbreaks [6].

The black soldier fly (BSF) *Hermetia illucens* has garnered significant attention from aquaculturists and researchers as an alternative to fishmeal. It has been partially or entirely incorporated into feed formulations for various cultured and ornamental fish, such as yellow catfish (*Pelteobagrus fulvidraco*) [7], European sea bass (*Dicentrarchus labrax)* [8], goldfish (*Carassius auratus)* [9], Nile tilapia [10], Atlantic salmon (*Salmo salar*) [11, 12], rainbow trout (*Oncorhynchus mykiss*) [13, 14], and pikeperch (*Sander lucioperca*) [15]. Despite the obvious benefits of fishmeal (FM) as an animal protein in aquafeeds, researchers are currently exploring novel options [16]. This is because of FM high price and decreased availability due to aquaculture development and the global economic crisis, which significantly affect commercial fish production.

BSF larvae meal is an eco-friendly protein source, offering a cost-effective method to convert organic waste into high-quality protein, ultimately increasing fish production [17, 18]. Abdel-Tawwab et al. [8] reported that partial **FM** replacement by BSF meal up to 50% reduced the feeding cost by 15.6% compared with the fishmeal control diet. *H. illucens* larval meal contains approximately 40–75% crude protein and 10–38% crude lipid, which relies on insect species and stage of life cycle, as well as has carotenoids and bioactive compounds such as chitin, lauric acid, and antimicrobial peptides, as the essential amino acids [19]. Fish dietary supplementation with black soldier larvae meal enhances growth, immunity, and disease resistance due to the immunostimulatory properties of chitin and chitosan polymers [10, 20]. The optimal dietary replacement levels of black soldier fly meal as a low-price feed ingredient and alternative to **FM** vary significantly among studies on fish species.

Nile tilapia, ranking third in tilapia production behind China and Indonesia, plays a crucial role as the primary cultured freshwater food species worldwide [21]. The production of Egyptian Nile tilapia has experienced rapid growth, comprising 56.63% of the total fish production by 2021 [22]. Sustainable development encounters various challenges, including high summer mortality and climate change. Furthermore, the elevated prices of feed ingredients are directly linked to ongoing war crises, impacting the government economy and food security [23, 24]. Despite the known benefits of defatted black soldier fly meal (DBSFM), its impact on the specific health parameters of Nile tilapia, especially in the context of bacterial infections, remains underexplored. This study aims to fill this gap by investigating the effects of DBSFM substituted diets on hematological, biochemical, immunological, and antioxidant activities and gene expression in Nile tilapia and the substitution’s protective role following *S. iniae* challenge.

## Materials and methods

### Fish acclimation and maintenance

Healthy Nile tilapia of average body weight (40.0 ± 0.50 g) was purchased from a private rearing farm at Kafr El-Sheikh Governorate, Egypt, transported to the Aquatic Animal Medicine wet Laboratory, Faculty of Veterinary Medicine, Benha University, Egypt, and acclimated in three indoor fiberglass circular tanks (500 L capacity) (90 fish/ tank) for two weeks with daily observation of fish health behavioral signs and lesions according to Austin et al. [25]. Fish were hand-fed with a commercial diet (30% crude protein, Aller Aqua, Egypt) two times daily (9 am and 4 pm) up to visual satiation.

### Experimental diets formulation

DBSFM was provided by EGYMAG Biotechnology Company, Egypt. A total of 3 diets with different replacement levels of FM by DBSFM as described elsewhere [26] were formulated as follows: 0% (control), 33%, or 100%, corresponding to 0, 66, or 200 g of DBSFM/ kg diet, respectively, to replace equivalent amounts of fishmeal (Table 1). All diet ingredients were ground (Table 1), mixed with oil and distilled water to form feeding dough, and pelleted with approximately 2.5 mm diameter using a meat mincer. Finally, the pellets were oven-dried at 50 °C for 24 h before being placed in a plastic bag and stored at 4 °C until use.

**Table 1:** Ingredients and proximate composition of experimental diets

Ingredients (g kg^− 1^)	Control diet	33%DBSFM	100%DBSFM	
Yellow corn	433.75	433. 73	433.71	
Soybean meal	290	290	290	
Defatted black soldier fly meal (DBSFM)	0	66	200	
Fish meal	200	134	0	
Vegetable oil	26	26	26	
Fish oil	24	24	24	
Molasses	20	20	20	
Vitamin and mineral premix	4	4	4	
Choline chloride	2	2	2	
Vitamin C	0.25	0.25	0.25	
D L methionine	00.000	0.02	0.04	
Total	1000	1000	1000	
Chemical analysis				
Dry matter	88.8	88.86	89.12	
Crud protein	29.82	29.78	29.76	
Crude fat	9.36	9.69	10.4	
Crude fiber	2.23	2.85	4	
Ash	8.22	8.09	7.74	
Gross energy kcal/kg diet	4325	4326	4326	
Lysine	1.96	1.95	1.95	
Methionine	0.7	0.7	0.7	
Chemical analysis of DBSFM				
Crud protein	55%			
Calcium	2.5%			
Phosphorus	1%			
Lysine	2.1%			
Methionine	0.9%			
Metabolism energy	2800 kcal/kg			

Vitamin premix supplied each kg of feed with Vitamin A = 7000 IU; Vitamin D = 1400 IU; Vitamin E = 10 mg; vitamin K3 3 mg; vitamin B1 1 mg; vitamin B2 4 mg; Vitamin B12 0.01 mg; Folic Acid 1 mg; Niacin 20 mg; Pantothenic acid 8 mg; Biotin 0.025; vitamin B6 1 mg; Copper 10 mg; Cobalt 0.01 mg; Iron 15 mg; Zinc 40 mg; Selenium 0.01 mg; Manganese 40 mg; Iodine 0.05 mg

### Bacteria strain

A virulent-verified strain of *S. iniae* (accession No MT086601) previously isolated from diseased gilthead seabream, *Sparus auratus* was gifted by the Fish Diseases Department, Faculty of Veterinary Medicine, Beni-Suef University, Egypt. The bacterial strain was grown in brain heart infusion broth (BHIB; HiMedia, India) and incubated at 28 °C for 24 h. The culture suspension was centrifuged at 3000 g/10 min. The supernatant was discarded, and the pellets were washed twice in phosphate-buffered saline at pH 7.4. The bacterial suspension’s optical density (OD) was adjusted to 1.3 at 600 nm, corresponding to 1 × 10^8^ cells/ml [27].

### Ethical statement

This study was performed in accordance with the guidelines of Animal Welfare and Research Ethics Committee of Benha University, Faculty of Veterinary Medicine, Egypt (BUFVTM 06-09-23).

### Rearing protocol

After acclimation, the fish were randomly distributed into nine tanks supplied with aerated and de-chlorinated tap water (30 fish/tank). Triplicate tanks were allocated to each of the three groups. Each group was hand-fed **on** of the prepared diets twice daily (9 am and 4 pm) at a rate of 4% of their body weight for six weeks (pre-challenge time). Upon completion of the feeding trial, ten fish from each tank/each group (control and supplemented) were transferred to a glass aquarium (30 × 40 × 60 cm) and allocated in triplicate (30 fish/group) for a challenging trial (2 weeks). Fish were injected intraperitoneally (IP) with a 0.1 ml *S. iniae* bacterial suspension (1 × 10^8^ cells/ml) [27]. During the post-challenge phase, the fish were maintained under identical conditions and feeding regimens as those employed in the pre-challenge phase. Partial water exchange (2/3) every other day using clean dechlorinated tap water to maintain good water quality parameters. Water quality parameters were measured by using a multiparameter bench photometer (HANNA^®^, HI83200-02, Schaumburg, IL, USA), and maintained within the optimal range for tilapia [28] and were as follows, temperature (27.20 ± 1.50 °C), dissolved oxygen (6.50 ± 0.50 mg/L) and pH (7.30 ± 0.10). The photoperiod was set to 12 h of light and 12 h of darkness for the duration of the experiment.

### Samples collection

Following the pre-challenge and post-challenge phases, fish were fasted for 24 h before sampling. Blood samples were collected from the caudal blood vessels of six fish from each group (two fish/ tank). The fish were sedated by immersion in buffered MS-222^®^ (Sigma-Aldrich) at a dose of 30 mg/L [29]. The blood samples were divided into two portions. One portion was transferred into Eppendorf tubes and mixed with EDTA as an anticoagulant for hematological and phagocytic activity. The other portion was left to clot at 4 °C and centrifuged at 3000 rpm for 10 min to obtain serum. The serum samples were collected and stored at − 20 °C for lysozyme activity and biochemical (total protein, albumin, cortisol, and glucose) analyses. Fish were then euthanasia (200 mg/L MS-222) [29], one at a time and dissected immediately. Liver and spleen samples (*n* = 3) were excised and processed for oxidant/antioxidant assays, in addition to other three liver samples were preserved in RNAlater^®^ (Sigma) for gene expression analysis.

### Hematological parameters

The total red blood cell (RBCs) count, hemoglobin (Hb), and packed cell volume (PCV) analyses were performed as previously described Anderson and Siwicki [30]. RBCs and white blood cells (WBCs) were counted using a hemocytometer and Natt–Herrik solution. Blood hemoglobin (Hb) concentration was determined using Drabkin’s colorimetric kit, and the absorbance was measured at 540 nm. Packed cell volume (PCV) was determined by microcentrifugation of fresh blood in a capillary tube (2500 rpm for 15 min at room temperature). Other blood indices (mean corpuscular volume (MCV), mean corpuscular hemoglobin (MCH), and mean corpuscular hemoglobin concentration (MCHC)) were estimated as described by Dacie and Lewis [31] as follows:

$$\:{\rm{MCV}}\,{\rm{(cm3/erythrocyte)}}\,{\rm{ = }}\frac{{PCV\:\left( \% \right)}}{{RBCs\:count\:}} \times \:10\:$$

$${\rm{MCH}}\,{\rm{(pg}}\,{\rm{Hb/erythrocyte)}}\,{\rm{ = }}\frac{{Hb}}{{RBCs\:count}} \times 10$$

$${\rm{MCHC}}\,{\rm{(g}}\,{\rm{Hb/100ml}}\,{\rm{erythrocytes)}}\,{\rm{ = }}\frac{{Hb}}{{PCV}} \times 100\:$$

The differential counts of lymphocytes, monocytes, neutrophils, basophils, and eosinophils were determined using prepared blood smears stained with Wright Giemsa according to Stoskopf [32].

### Biochemical and immunological assay

Serum total protein and albumin levels were measured using commercially available kits (Biodiagnostics, Egypt). The globulin level was calculated by subtracting the albumin level from the total protein concentration. Commercial test kits (Biodiagnostic, Egypt) were used in the current study to determine glucose and cortisol concentrations in serum samples, following the manufacturer’s instructions.

Serum lysozyme activity was measured as described by Ellis [33], using *Micrococcus lysodeikticus* (Sigma Chemical Co.), and the unit of lysozyme present in the serum (μg/ml) was obtained from a standard curve made with lyophilized hen-egg-white-lysozyme (Sigma). The phagocytic activity was determined according to the method described by Kawahara et al. [34]. The number of phagocytosed cells was counted to calculate phagocytic activity according to the following equation:

$${\rm{Phagocytic}}\,{\rm{activity}}\,{\rm{(\% )}}\,{\rm{ = }}\:\frac{{{\rm{macrophages}}\:{\rm{containing}}\:{\rm{yeast}}\:}}{{{\rm{total}}\:{\rm{number}}\:{\rm{of}}\:{\rm{macrophages}}\:}} \times 100$$

### Oxidant and antioxidant analysis

Liver and spleen tissues (one gram) (*n* = 3) were homogenized in cooled phosphate-buffered saline (pH 7.4) at a ratio of 1:10 (w/v) using an electrical homogenizer (Heidolph, Germany) [35]. The supernatants were obtained after centrifugation of the homogenates at 4 °C at 3000 ×g for 15 min and stored at–20 °C for oxidant/ antioxidant enzyme analysis.

Total antioxidant capacity (TAC) was determined as described by Koracevic and Koracevic [36] and expressed as mM/L. While, the lipid peroxide (malondialdehyde) (MDA) content as nmol/g tissue, was assessed at 534 nm [37]. Superoxide dismutase (SOD) level was measured at 560 nm by the enzymatic colorimetric method [38], catalase (CAT) level was assayed by measuring the reduction of hydrogen peroxide concentration at 240 nm and it is expressed as U/g tissue [39], and glutathione peroxidase (GSH-Px) was assayed via a coupled assay with glutathione reductase by measuring the rate of NADPH oxidation at 340 nm using H_2_O_2_ as the substrate and expressed as U/g tissue [40]. All assays were measured using a diagnostic commercial kit (Biodiganostic Company, Cairo, Egypt).

### RNA extraction, cDNA synthesis, and gene expression analyses

Total RNA was manually extracted from 100 mg of each liver sample from each group using a handheld homogenizer to homogenize the tissue immersed in one **ml** Genzol™ (Geneaid Biotech Ltd, Taiwan) without DNase treatment, and the pellet was dissolved in TE buffer (pH 8.0), as described previously [41]. RNA quantity was determined using a NanoDrop spectrophotometer (Q5000/Quawell, Massachusetts, USA). cDNA was synthesized using 1 μg of total RNA with the TOPscript™ RT DryMIX (dT18) cDNA Synthesis Kit (Enzynomics Co Ltd, Daejeon, Republic of Korea), following the manufacturer’s protocol. Specific primers were used to amplify genes of interest in Nile tilapia, including the pro-inflammatory genes tumor necrosis factor-alpha (*TNF-α*) and interleukin 1 beta (*IL-1β*), as well as the anti-inflammatory genes transforming growth factor-β (*TGF-β*) and interleukin-10 (*IL-10)*. β-Actin was used as the housekeeping gene. Detailed information regarding the primers used is available elsewhere [42]. The QuantStudio™ 1 Real-Time PCR System (Applied Biosystems™, Thermo Fisher Scientific, USA) was used to measure gene expression using the Solg™ 2X Real-Time PCR Smart Mix (including SYBR^®^ Green) (SolGent Co., Ltd., Yuseong-gu, Daejeon, Korea). The thermocycling conditions for the reaction were as follows: 95 °C for 20 s, followed by 40 cycles of denaturation at 63 °C for 40 s, and elongation at 72 °C for 30 s. The mRNA expression data were standardized to *β*-Actin using the 2^−ΔΔCT^ method [43].

### Statistical analysis

Data was analyzed using GraphPad Prism version 8 (GraphPad Software Inc.) and subjected to normality and homogeneity of variance tests using the Kolmogorov-Smirnov and Levene’s tests. Two-way ANOVA was then used to determine the differences between groups with respect to time, treatment dose, and combined effects of treatment dose and time. Tukey’s and Bonferroni’s multiple comparison tests were used to assess the differences between replicate means of the groups before and after the challenge. The significance level was set at *P <* 0.05 (*), *P <* 0.01 (**), *P <* 0.001 (***).

## Results

### Hematological parameters

**Fig. 1:**
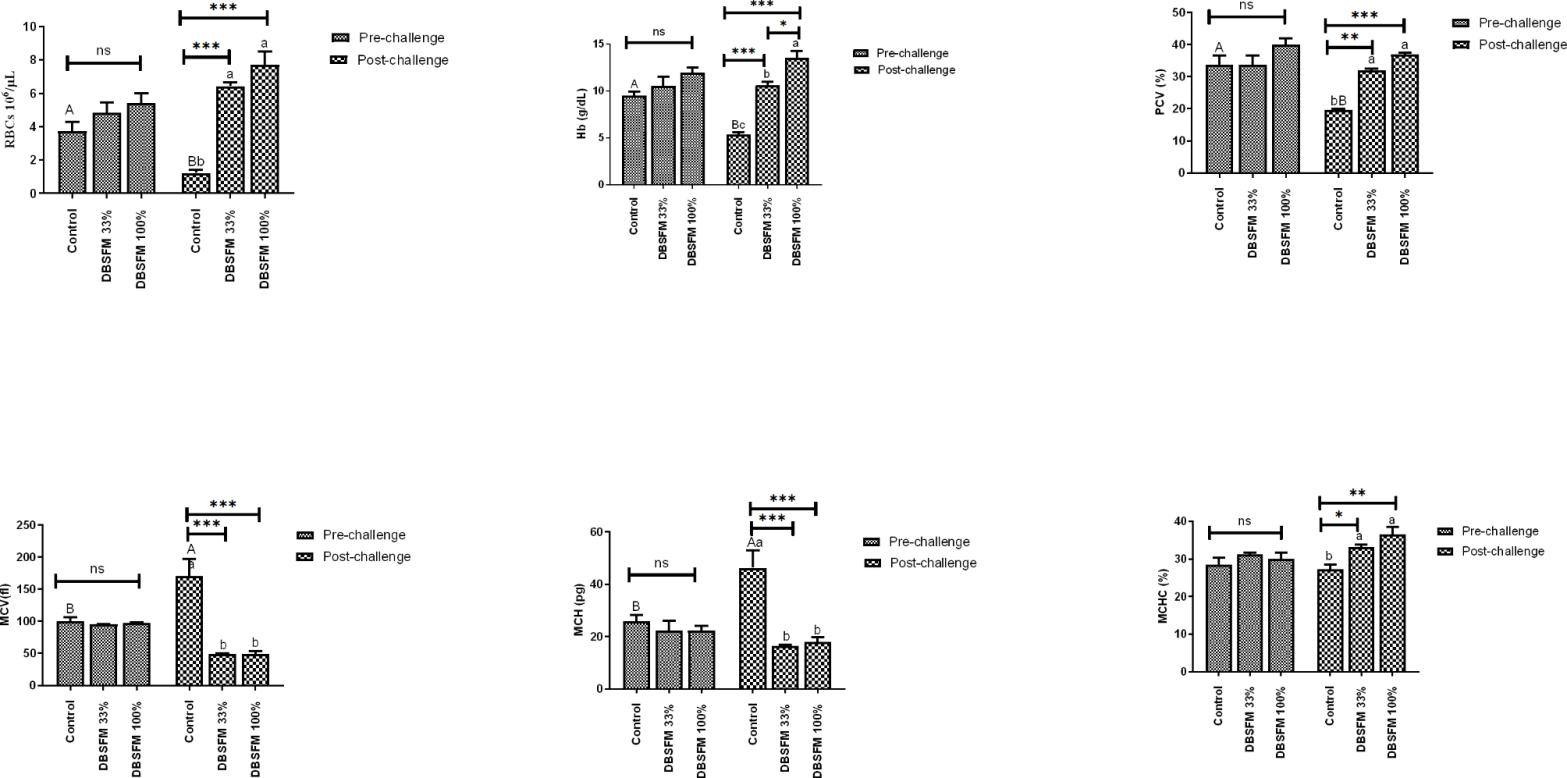
Pre- and post-challenge hemogram in Nile tilapia fed 0%, 33%, and 100% of DBSFM-supplemented diets. Data were expressed as the mean of six fish ± SEM. Values with a different letter superscript significantly differ between groups within the same period. Values with a different capital letter superscript significantly differ between the same groups pre- and post-challenge. Significant levels (*P* < 0.05, 0.01, and 0.001), as determined by Two-way ANOVA

The effects of the DBSFM substitution on the hemogram are presented in Fig. 1. After the pre-challenge period, there were no statistically significant changes in the hemogram between the groups. The post-challenge group showed substantial increases in RBCs, Hb concentration, PCV, and MCHC in the DBSFM-treated groups compared to the control group. However, MCV and MCH were significantly decreased (*P* < 0.001) in the DBSFM-treated groups compared with the control. Upon comparing the pre- and post-challenge groups, it was observed that the RBCs of the control group demonstrated a significant decrease post-challenge (*P* < 0.05), along with significant decreases (*P* < 0.01) in Hb and PCV compared to their pre-challenge levels. An opposite trend was observed for MCV and MCH. The time*group interaction significantly affected the hemogram picture.

**Fig. 2:**
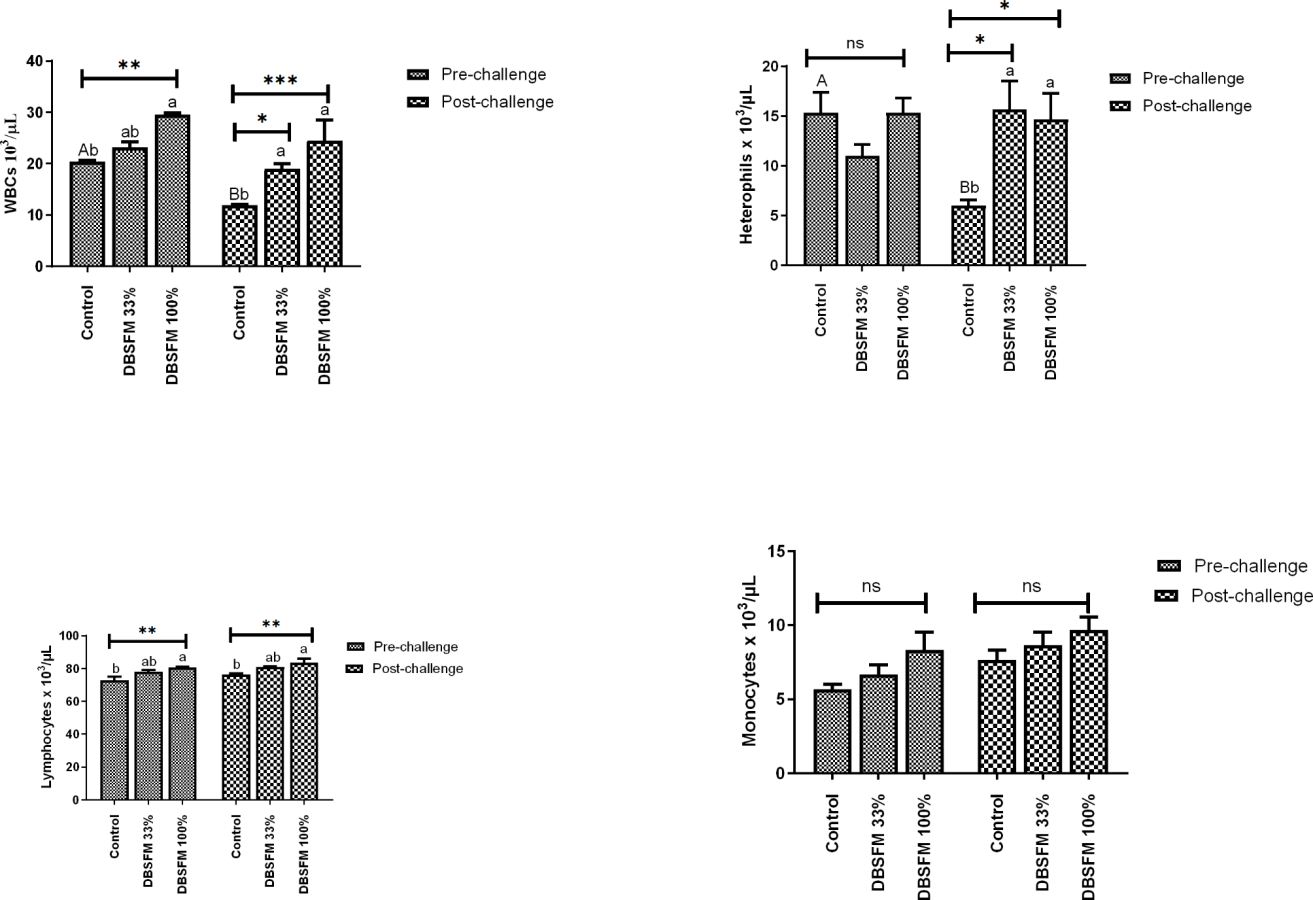
Pre- and post-challenge leukogram in Nile tilapia fed 0%, 33%, and 100% of DBSFM-supplemented diets. Data were expressed as the mean of six fish ± SEM. Values with a different letter superscript significantly differ between groups within the same period. Values with a different capital letter superscript significantly differ between the same groups pre- and post-challenge. Significant levels (*P* < 0.05, 0.01, and 0.001), as determined by Two-way ANOVA

The effects of the DBSFM substitution on the leukogram are presented in Fig. 2. Including DBSFM in the Nile tilapia diet led to improved WBCs and lymphocyte counts in the DBSFM-100% group, especially after the pre-challenge period (*P* < 0.01). Post-challenge, WBCs, lymphocyte, and heterophil counts significantly increased in **DBSFM**-treated groups compared to the control. Upon comparing the pre-and post-challenge groups, it was observed that the control group’s WBCs and heterophil counts demonstrated a significant decrease in the post-challenge phase (*P* < 0.05). The time*group interaction did not significantly affect the leukogram picture.

### Biochemical and immunological parameters

**Fig. 3:**
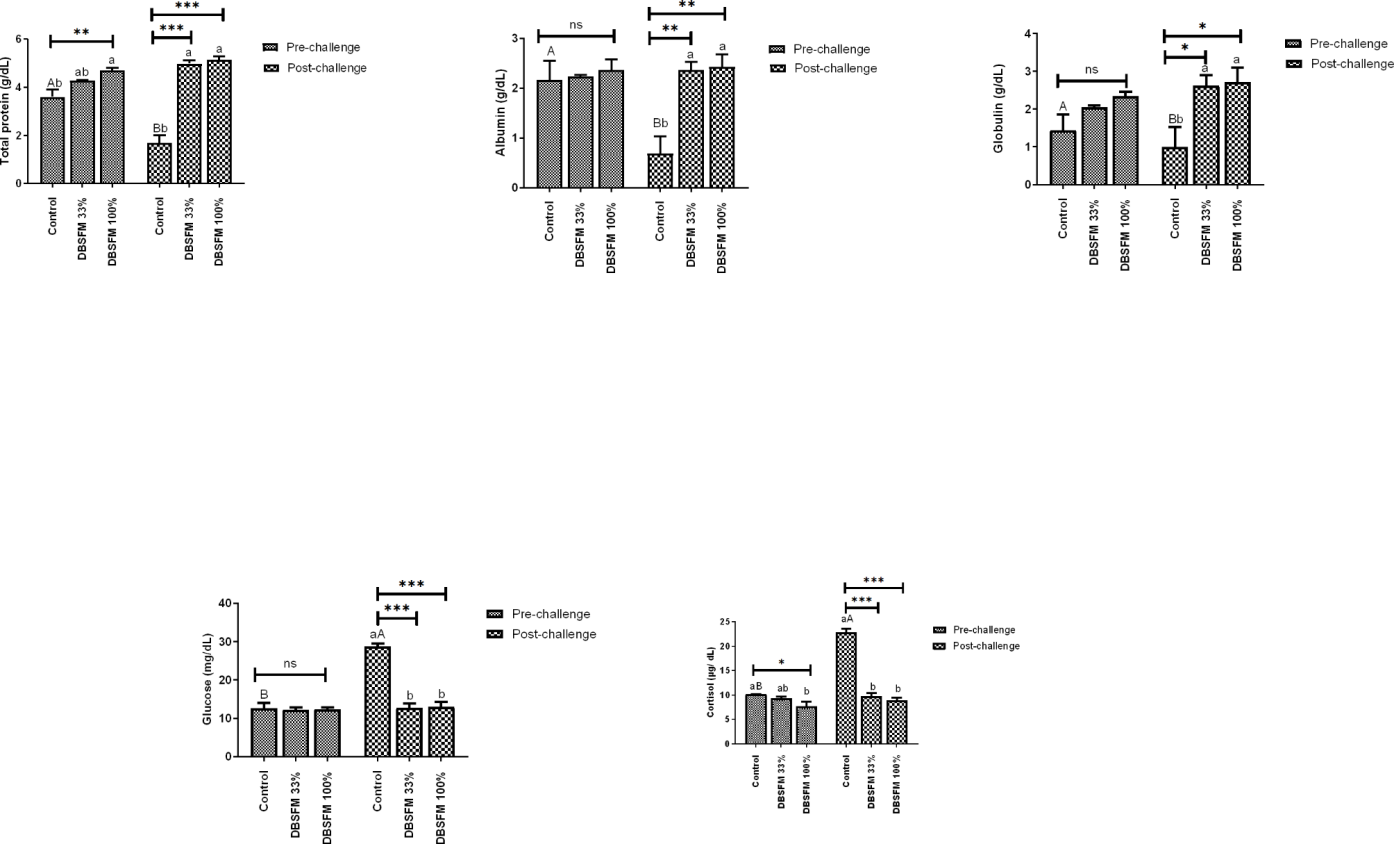
Pre- and post-challenge biochemical parameters in Nile tilapia fed 0%, 33%, and 100% of DBSFM-supplemented diets. Data were expressed as the mean of six fish ± SEM. Values with a different letter superscript significantly differ between groups within the same period. Values with a different capital letter superscript significantly differ between the same groups pre- and post-challenge. Significant levels (*P* < 0.05, 0.01, and 0.001), as determined by Two-way ANOVA

The DBSFM-100% group showed a significant improvement in total protein levels (*P* < 0.01) after the pre-challenge period, whereas there were no notable differences in albumin and globulin levels among the groups. Post-challenge, the **DBSFM**-treated groups exhibited increased levels of total protein, albumin, and globulin (*P* < 0.001, *P* < 0.01, and *P* < 0.05, respectively) compared with the control group. When comparing the groups pre- and post-challenge, there was a significant decrease (*P* < 0.01) in the control group’s levels post-challenge compared to their respective levels before the challenge (Fig. 3). The time*group interaction significantly affected the protein profile.

Following the pre-challenge period, it was observed that DBSFM did not have a significant effect (*P* > 0.05) on glucose levels. In contrast, the cortisol level significantly decreased only in the **DBSFM**-100% group compared to other groups. Glucose and cortisol levels displayed similar patterns at post-challenge, where their levels declined dramatically (*P* < 0.001) in the **DBSFM**-treated groups compared to the control group. Concerning the groups’ comparison of pre-and post-challenge, there was a significant increase (*P* < 0.001) in the control group’s levels of glucose and cortisol post-challenge compared with their respective pre-challenge levels (Fig. 3). The time *group interaction significantly influenced glucose and cortisol levels.

**Fig. 4:**
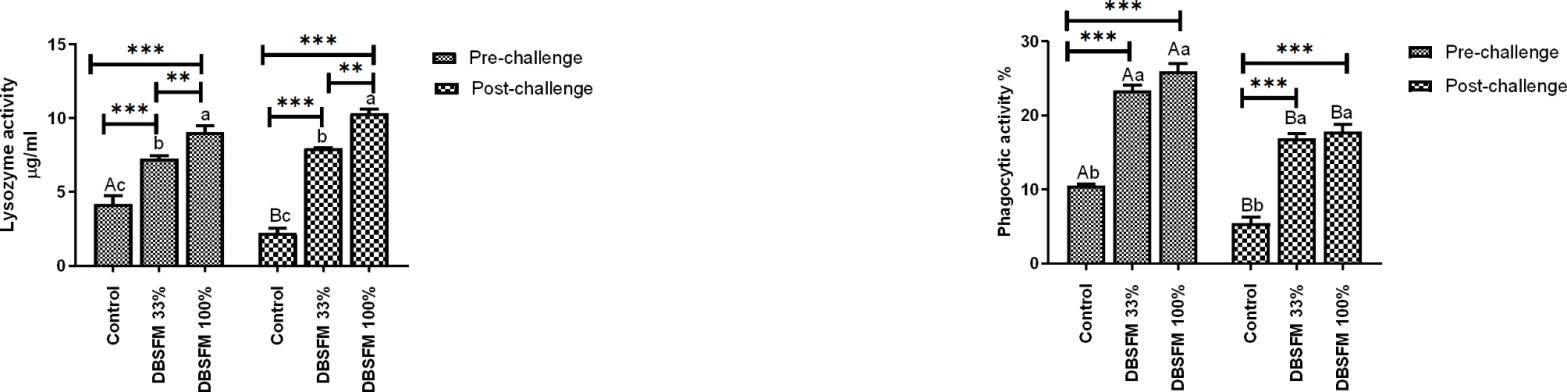
Pre- and post-challenge lysozyme and phagocytic activity in Nile tilapia fed 0%, 33%, and 100% of DBSFM-supplemented diets. Data were expressed as the mean of six fish ± SEM. Values with a different letter superscript significantly differ between groups within the same period. Values with a different capital letter superscript significantly differ between the same groups pre- and post-challenge. Significant levels (*P* < 0.05, 0.01, and 0.001), as determined by Two-way ANOVA

Lysozyme and phagocytic activities displayed a similar trend, where their levels increased significantly (*P* < 0.001) among the DBSFM-treated groups after the pre- and post-challenge phases compared to the control. Comparing lysozyme activity of pre- and post-challenge phases revealed that lysozyme activity was significantly decreased in control group post-challenge compared to their pre-challenge level. While phagocytic activity showed a substantial decline in all groups post-challenge compared to their pre-challenge counterparts (Fig. 4). The time*group interaction significantly affected lysozyme activity but had no significant effect on phagocytic activity.

### Oxidant and antioxidant parameters

**Fig. 5:**
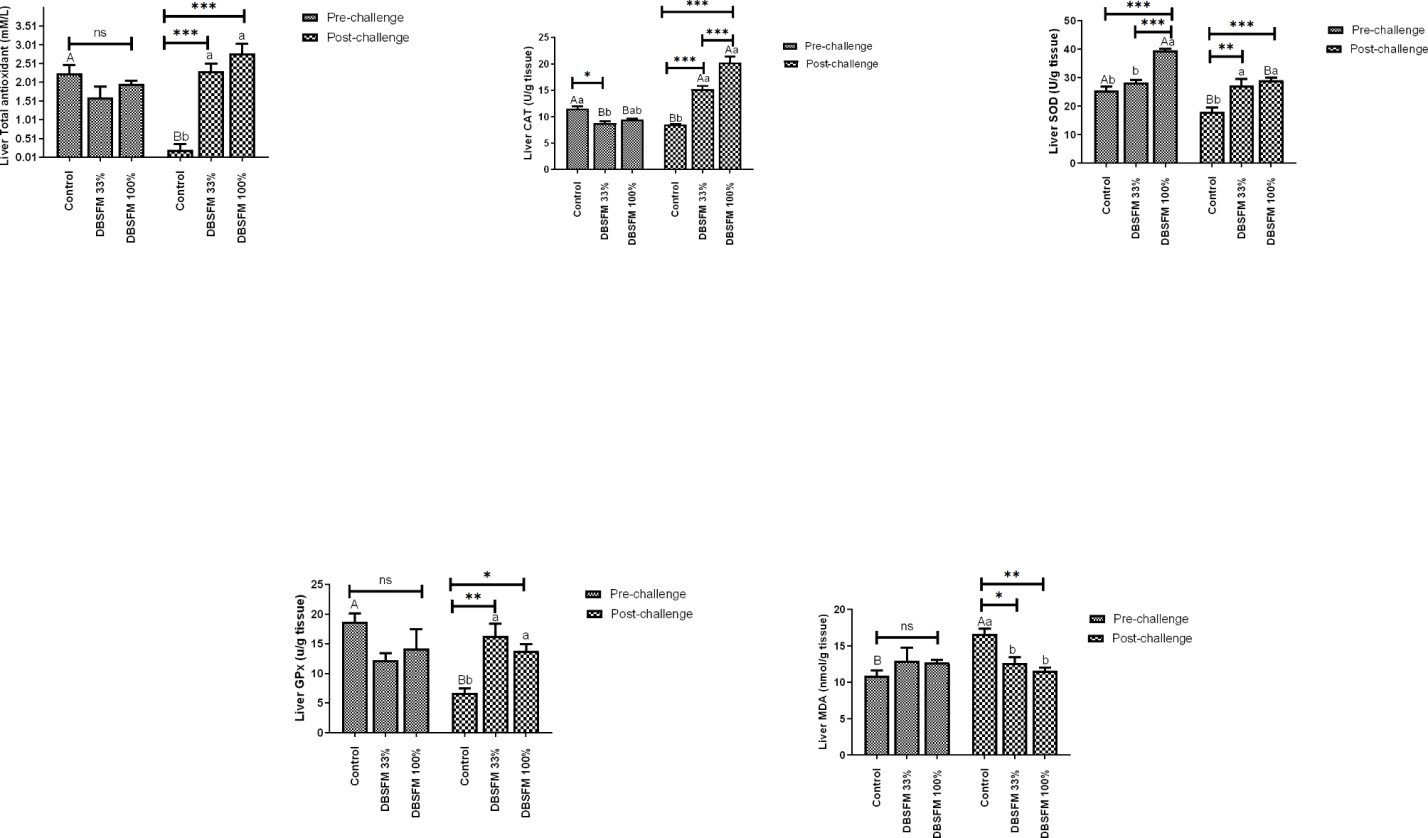
Pre- and post-challenge of total antioxidant capacity (TAC), catalase (CAT), superoxide dismutase (SOD), glutathione peroxidase (GPx), and malondialdehyde (MDA) levels in the liver of Nile tilapia fed 0%, 33%, and 100% of DBSFM-supplemented diets. Data were expressed as the mean of six fish ± SEM. Values with a different letter superscript significantly differ between groups within the same period. Values with a different capital letter superscript significantly differ between the same groups pre- and post-challenge. Significant levels (*P* < 0.05, 0.01, and 0.001), as determined by Two-way ANOVA

**Fig. 6:**
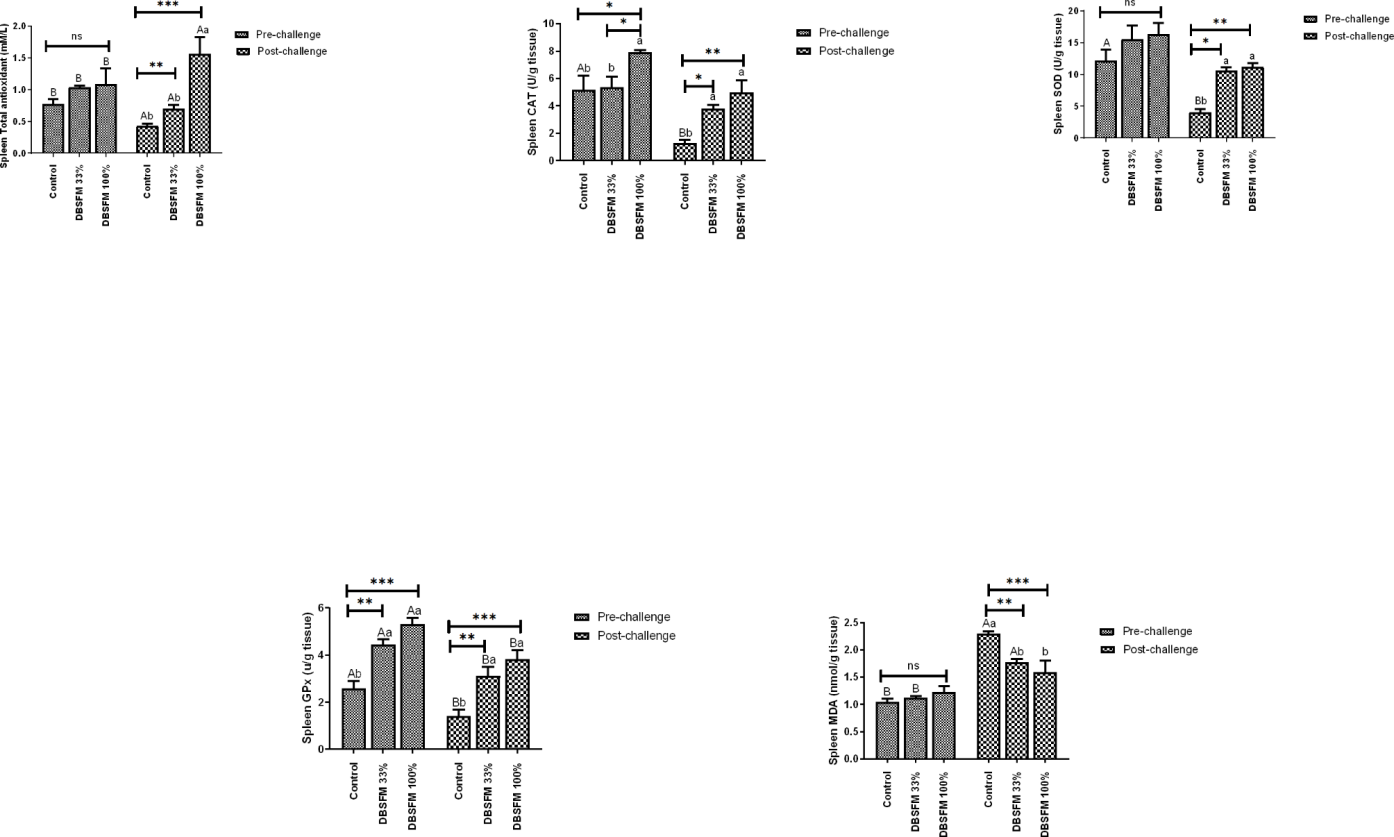
Pre- and post-challenge of total antioxidant capacity (TAC), catalase (CAT), superoxide dismutase (SOD), glutathione peroxidase (GPx), and malondialdehyde (MDA) levels in the spleen of Nile tilapia fed 0%, 33%, and 100% of DBSFM-supplemented diets. Data were expressed as the mean of six fish ± SEM. Values with a different letter superscript significantly differ between groups within the same period. Values with a different capital letter superscript significantly differ between the same groups pre- and post-challenge. Significant levels (*P* < 0.05, 0.01, and 0.001), as determined by Two-way ANOVA

The effects of DBSFM substitution on oxidant/antioxidant variables in the liver and spleen are presented in Figs. 5 and 6. TAC in the liver and spleen following the pre-challenge phase showed no significant differences (*P* > 0.05) among the groups. Post-challenge, liver TAC displayed a highly significant enhancement (*P* < 0.001) in the **DBSFM**-treated groups compared to the control. Meanwhile, Splenic TAC showed considerable improvement in the **DBSFM**-100% group compared to the control group (*P* > 0.001) and the **DBSFM-**33% group (*P* > 0.01).

Following the pre-challenge period, MDA levels in the liver and spleen were not significantly different (*P* > 0.05) between the groups. Post-challenge, MDA levels in the liver and spleen were significantly decreased in the **DBSFM**-treated groups compared to the control. When comparing the groups before and after the post-challenge, a significant increase (*P* < 0.05) in hepatic MDA level was noted in the control group post-challenge compared to its respective level before the challenge. While the Splenic MDA showed a significant increase in the control (*P* < 0.01) and DBSFM-33% (*P* < 0.05) groups post-challenge compared to their respective levels before the challenge.

With respect to the SOD activity, it was observed that hepatic SOD showed a significant increase (*P* < 0.001) in the **DBSFM**-100% group compared to other groups following the pre-challenge period. In contrast, splenic SOD activity displayed no significance among groups. Post-challenge, SOD activity displayed a similar pattern, where its activity increased in **DBSFM**-100% groups (Hepatic SOD, *P* < 0.001, and splenic SOD, *P* < 0.01) and **DBSFM**-33% (Hepatic SOD, *P* < 0.01, and splenic SOD, *P* < 0.05) groups compared to the control.

The hepatic CAT activity was moderately decreased (*P* < 0.05) in the **DBSFM**-33% group compared with the control group. While splenic CAT showed a significant increase (*P* < 0.05) in the **DBSFM**-100% group compared with other groups following the pre-challenge period. Post-challenge, hepatic **CAT** showed a significant increase (*P* < 0.001) between the **DBSFM**-treated and control groups. Similarly, the splenic CAT revealed increased activity in **DBSFM**-treated groups (**DBSFM**-100%, *P* < 0.01, and **DBSFM**-33%, *P* < 0.05) compared to the control.

The hepatic GPx activity showed no significant changes among the groups (*P* > 0.05), while the splenic GPx showed increased activity in **DBSFM**-treated groups (**DBSFM**-100%, *P* < 0.001, and **DBSFM**-33%, *P* < 0.01) compared to the control following the pre-challenge period. Post-challenge, the hepatic and splenic GPx activities increased in **DBSFM**-treated groups (**DBSFM**-100%, *P* < 0.05, *P* < 0.001, and **DBSFM**-33%, *P* < 0.05) compared to the control group.

Concerning the group comparison pre- and post-challenge, a significant decrease (*P* < 0.05) in hepatic CAT and SOD activities and in hepatic GPx and TAC (*P* < 0.01) was noted in the control group post-challenge compared to their respective pre-challenge levels. Additionally, splenic TAC, CAT, and SOD showed a significant decrease (*P* < 0.05) along with GPx (*P* < 0.01) in the control group post-challenge compared to their respective levels’ pre-challenge. Similarly, the **DBSFM**-treated groups showed different changes either at both doses or at only one dose, as depicted in Figs. 4 and 5. It is worth noting that the time*group interaction significantly affected the levels of hepatic CAT (two-way ANOVA, *P* < 0.001), SOD, GPx, TAC (two-way ANOVA, *P* < 0.01), and MDA (two-way ANOVA, *P* < 0.05). The time*group interaction significantly influenced the splenic TAC (two-way ANOVA, *P* < 0.01) and MDA (two-way ANOVA, *P* > 0.05), with no significant effects on CAT, SOD, and GPx.

### Genes expression analyses

**Fig. 7:**
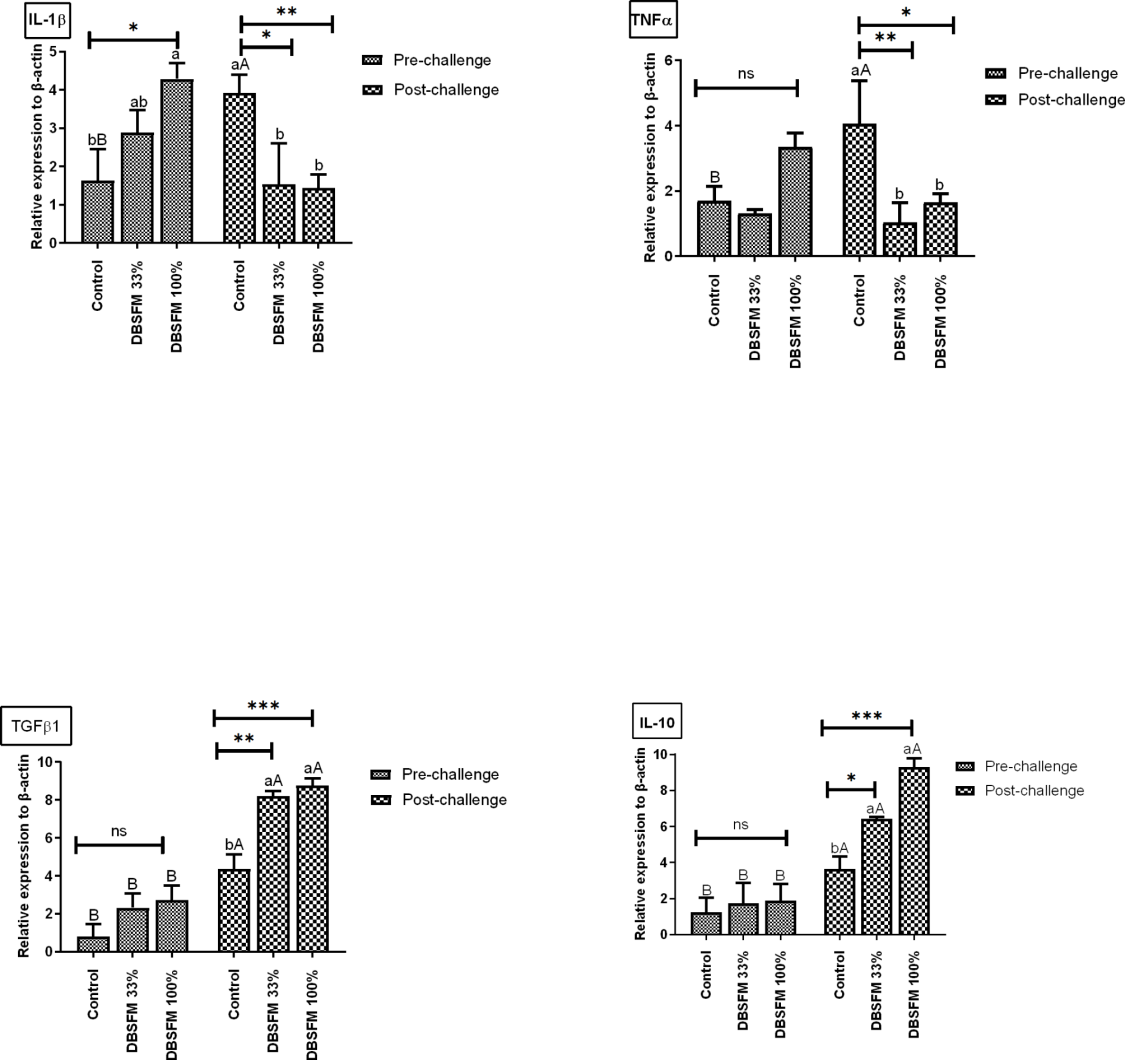
Pre- and post-challenge gene expression levels in the liver of Nile tilapia fed 0%, 33%, and 100% of DBSFM-supplemented diets. Data were expressed as the mean of six fish ± SEM. Values with a different letter superscript significantly differ between groups within the same period. Values with a different capital letter superscript significantly differ between the same groups pre- and post-challenge. Significant levels (*P* < 0.05, 0.01, and 0.001), as determined by Two-way ANOVA

The effect of DBSFM dietary replacement on pro- (*TNF-α* and *IL-1β*) and anti-inflammatory (*TGF-β* and IL-10) gene expression in the liver is presented in Fig. 7. Following the pre-challenge phase, there were no statistical changes in all gene expression levels, except for the *IL-1β*, which showed a significant up-regulation (∼ 3-fold change, *P* < 0.05) in the **DBSFM**-100% group compared to the control. Following the post-challenge phase, *IL-1β* and *TNF-α*, exhibited a significant decrease in their expression levels ∼ 3-fold change in the BSF-groups (*IL-1β*, **DBSFM**-100%, *P* < 0.01; and DBSFM-33%, *P* < 0.05; *TNF-α*, DBSFM-100%, *P* < 0.05; and DBSFM-33%, *P* < 0.01) compared to the control group. The opposite trend was observed for the *TGF-β* and *IL-10*, where they showed a significant increase ∼ 3-fold change (*P* < 0.01) in the DBSFM-100% group and DBSFM-33% (*TGF-β1*, DBSFM-33%, *P* < 0.01; *IL-10*, DBSFM-33%, *P* < 0.05) compared to the control group.

When comparing the groups between the pre- and post-challenge phases, it was noted that pro-inflammatory cytokines (*IL-1β* and *TNF-α*) increased significantly ∼ 3-fold change (*P* < 0.01) in the control group post-challenge compared to their respective pre-challenge level. The anti-inflammatory cytokine (*TGF-β* and *IL-10)* increased significantly in control ∼ 3-fold change (*P* < 0.01) and DBSFM-groups ∼ 4-fold change (*P* < 0.001) post-challenge compared to their respective levels’ pre-challenge. It is worth noting that the time*group interaction significantly affected *IL-10* expression levels, with no significant effects on other gene expression levels.

## Discussion

Hematological indices are crucial for evaluating the overall health and physiological stress in fish [44]. In the present study, RBCs, Hb concentration, PCV, and blood cell indices (MCV, MCH, and MCHC) in all the pre-challenged groups showed no significant changes with DBSFM inclusion in the Nile tilapia diet. These results are coincided with previous studies on goldfish [44], European seabass [8], African catfish [45], and Nile tilapia [10].

As far as the authors can ascertain, this is the first study to evaluate a comprehensive array of selected parameters after the time (two weeks) post-challenge with *S. iniae*, contrary to other studies evaluating the BSF-substituted effects shortly after post-challenge with biotic or abiotic factors. The Nile tilapia-fed *Zophobas morio* meal showed an increase in erythrocyte counts at 6 h post-challenge with *Escherichia coli* (*E. coli*) lipopolysaccharides (LPO) [46]. This study proposes that including **DBSFM** in the diets of Nile tilapia enhances the blood profile and reduces the negative impacts of *S. iniae*. The decrease in hematological parameters observed in the control groups following the challenge, as compared to the pre-challenge period, is likely due to the bacteria’s propensity to cause hemolysis of red blood cells [47]. In the current study, WBCs counts, lymphocytes, and heterophils displayed increments in pre- and post-challenge periods, suggesting that fish fed DBSFM mobilized circulating WBCs more rapidly than fish fed a control diet to combat infection. Neutrophils are the first inflammatory cells recruited to the infection [48]. This increase in these immune cells stimulates the body’s immune system to react against bacterial challenges. Consistently, Nile tilapia fed with insect meal *Zophobas morio* showed a significant increase in WBCs, thrombocytes, and neutrophils after a 6 h challenge with *E. coli* lipopolysaccharide [46].

The total protein level in the current study demonstrated significant improvement in the group fed a 100% DBSFM inclusion diet at the pre-challenge time. Kamalii et al. [9] reported that dietary protein replacement of fish meal with black soldier fly larvae meal up to 60% (201 g/ kg) in goldfish diet considerably increased total protein, albumin, and globulin values. In contrast, Abdel-Tawwab et al. [8] and Lu et al. [49] reported that total protein, albumin, and globulin were not significantly affected by dietary BSFM supplementation in European sea bass, grass carp (*Ctenopharyngodon idellus*), and goldfish, respectively.

With regard to the post-challenge period, the total protein, albumin, and globulin levels of Nile tilapia-fed **DBSFM** were significantly increased in all treated groups compared with the challenged control group. Meanwhile, a study by Chaklader et al. [50] reported no significant interaction between pre-challenge and 24 h post-challenge juvenile barramundi fed 60% and 75% poultry by-product (PBM) supplemented with 10% BSFM. Increased total protein and albumin levels indicate an improvement in the fish immune system [51], especially after bacterial challenge, as they are responsible for the transportation of biological substances, such as enzymes, vitamins, and hormones [52]. Upon comparing the pre-and post-challenge groups, there was a notable decline in the immunological response of the control group after the challenge relative to their levels before the challenge, which indicates liver and kidney damage by the invasion of *S.iniae* or toxins, leading to a notable decrease in the serum proteins synthesis in the liver and an increase in protein loss due to renal degeneration [53].

Cortisol is the main glucocorticoid hormone emitted in response to stress and has been used as a marker of various environmental stressors [54]. Cho et al. [55] reported no significant difference in blood cortisol levels between the rainbow trout group fed BM reared under high densities (9.8 kg/m^3^) and the control group. As observed herein, no significant effect had been noted on glucose and cortisol levels; only the **DBSFM**-100% group displayed a significant decrease in cortisol levels compared to the control following the pre-challenge period, which indicates that dietary replacement of fishmeal with BSF meal did not influence these stress biomarkers. Fatima et al. [56] reported that substituting soybean meal with black soldier fly larvae meal up to 80% and 100% in juvenile rohu (*Labeo rohita*) significantly decreased glucose levels at the end of 8 weeks of feeding experiment. Additionally, partial replacement of FM with DBSF in the gilthead seabream diet did not affect glucose, cortisol, or total protein levels after 131 days of feeding trials [57]. The **DBSFM**-treated groups showed a significant decrease in glucose and cortisol concentrations after the *S. iniae* challenge, which triggered the fish immune response as observed in **DBSFM**-supplemented groups. Under biological stress such as bacterial infection, cortisol hormone and glucose are produced to manage the stress and inflammation caused by the pathogen. **DBSFM** could help in modulating stress response by improving overall health conditions through its bioactive compounds such as antimicrobial peptides and fatty acids, resulting in lower cortisol and glucose concentrations after the infection.

The innate immune system is the primary defense tool and the primary defense mechanism in fish [58]. Lysozymes play a major role in fighting fish diseases through the breakdown of 1,4 glycosidic bonds found in the peptidoglycan cell walls of both Gram-positive and Gram-negative bacteria [59]. Lysozyme activity following the pre-challenge phase significantly increased in groups fed DBSFM-substituted diets, which could be attributed to the increased number of blood neutrophils [60]. Similarly, lysozyme activity in the skin mucus of Nile tilapia fed 4%, and 6% BSFLM substituted diets was significantly stimulated after 12 weeks of feeding [10]. The plasma lysozyme activity of rainbow trout [61] and European sea bass [8, 62] was significantly enhanced by dietary black soldier fly substitution.

In the post-challenge period, dietary inclusion of **DBSFM** improved the lysozyme activity of Nile tilapia compared to the control. Chaklader et al. [63] reported that barramundi, *Lates calcarifer* fed 45% poultry by-product supplemented with 10% black soldier fly meal significantly improved lysozyme activity challenge with *Vibrio harveyi*. Plasma lysozyme activity in Nile tilapia-fed enriched black soldier fly larval meal supplemented with chitinase significantly increased after a 24 h intraperitoneal injection of *E. coli* lipopolysaccharide [64]. Our findings suggest that DBSFM-enhanced lysozyme activity may be due to the presence of chitin in BSFM, which augments gut microbiota and, hence, fish immunity [61, 65]. Moreover, the immunostimulatory effect of an insect-based meal may be attributed to antimicrobial peptides secreted by insects [66].

Cytokines mediate the phagocytic activity of neutrophils and macrophages [67], which ingest and kill bacteria primarily by producing reactive oxygen species (ROS) during a respiratory burst. The present study showed that DBSFM substitution in Nile tilapia diets significantly increased phagocytic activity pre- and post-challenge. Similarly, Abdel-Latif et al. [62] showed that BSFLM considerably improves phagocytic activity in European sea bass. It has been reported that black soldier flies have antimicrobial activity; therefore, they may be the best alternative to antibiotics [68]. Chito-oligosaccharides (GlcNAc4) are produced from the hydrolysis of chitin of BSF by the chitinase enzyme; this product has shown immune-enhancing, anti-inflammatory, and antimicrobial resistance [69]. Similarly, the dietary inclusion of houseflies (*Musca domestica*) in the red sea bream diet enhanced phagocytic activity [70]. Opposite to the present findings, phagocytic activity significantly decreased in yellow catfish-fed fishmeal replacement diets with 85% and 100% BSF for 65 days [7]. The variability of the results throughout the research can be attributed to factors like fish species, size, experimental setting, feeding duration, type of insect meal, and level of replacement.

Total antioxidant capacity (TAC) is the collective measurement of all antioxidants present in an organism’s cells, indicating the overall level of enzymatic and non-enzymatic antioxidants [71]. In the current study, TAC in the liver and spleen following the pre-challenge phase exhibited no significant differences (*P* > 0.05) in the **DBSFM**-treated groups compared with the control. Similarly, the replacement of FM by BSFLM up to 75% in the Jian carp diet revealed no significant effect on total antioxidant capacity, but 100% BSFLM replacement showed a significant decrease in TAC [72]. In addition, dietary inclusion of DBSFM at 1% and 2% for 62 days in largemouth bass, *Micropterus salmoides* showed no increase in TAC [73]. In the present study, the splenic TAC showed a noteworthy enhancement after post-challenge, particularly in the **DBSFM**-100% group. Nevertheless, the liver’s TAC showed a substantial improvement in both the **DBSFM**-100% and 33% groups compared to the control, which coincided with the TAC pattern in a study conducted by Zahran et al. [74].

Fish antioxidant defense systems mainly consist of antioxidant enzymes responsible for diminishing **ROS** which can induce lipid peroxidation and DNA and protein damage [75]. Superoxide anions are first converted to hydrogen peroxide (H_2_O_2_) by SOD, and H_2_O_2_ is then catalyzed into water and molecular oxygen by CAT-GPx [76]. MDA is a product of polyunsaturated fatty acid peroxidation and directly reflects the level of lipid peroxidation [77]. In the present study, MDA levels in the liver and spleen were not significantly different after the pre-challenge period. Consistently, dietary supplementation with graded levels of defatted BSF in juvenile Jian carp juveniles [78] and snakehead (*Channa striata*) juveniles [79] showed no significant differences in serum or hepatic MDA levels. Decreased serum MDA concentrations were reported by Zhou et al. [72] in Jian carp and Japanese seabass-fed BSFLM.

In the post-challenge period, MDA levels in the liver and spleen were significantly decreased in the **DBSFM**-treated groups. Furthermore, rohu and catla (*Catla catla*) fed BSF substituted diets showed a significant decrease in MDA levels 15 days post-challenge with *Staphylococcus aureus* [56]. As observed, the decreased MDA levels in Nile tilapia challenged with *S. iniae* indicated an increased antioxidant enzyme system upon DBSFM replacement, which could protect fish against ROS production.

In this study, the hepatic SOD and splenic CAT activities were significantly enhanced in the **DBSFM**-100% group after the pre-challenge period. CAT activity was significantly increased in Jian carp [78] and mirror carp [80] fed BSFM. Moreover, SOD and CAT activities were significantly increased in rohu and catla [56], and European seabass [62] that received BSFM for 8 and 60 days, respectively. Conversely, Elia et al. [13], Wang et al. [81], and Jiang et al. [82] demonstrated no significant differences in SOD and CAT activities in the liver and serum of rainbow trout, Japanese seabass, and juvenile groupers (*Epinephelus coioides*) fed BSFM.

With respect to the post-challenge period, SOD and CAT activities increased in the BSF-treated groups compared to the control. To the best of our knowledge, only a few studies have reported the effect of dietary inclusion of BSF or other insect meal on fish oxidative response post-stress. Fatima et al. [56] observed that SOD and CAT increased with an increase in the proportion of BSFLM in the diet of rohu and catla challenged with *Staphylococcus aureus.* This increase in the SOD and CAT levels is probably due to the BSF substitution and its chitin content, which has scavenging activity against free radicals to overcome ROS production induced by the bacterial challenge [83, 84]. Splenic GPx activity was enhanced, whereas hepatic GPx activity showed no significant differences among the treated groups at pre-challenge. Similarly, GPx activity in the liver was not affected, whereas it was significantly elevated in the plasma of striped snakehead juveniles that received a BSF diet [79]. Juvenile European seabass showed significant elevation in hepatic GPx activity following BSF dietary supplementation for 60 days [55]. Conversely, in the present study, splenic and hepatic GPx activity significantly increased post-challenge in all groups that received DBSFM-substituted diets. TAC, CAT, and GPx were not affected by dietary insect meal *Tenebrio molitor* larvae after air exposure challenge in European sea bass, except for SOD, which showed a significant increase post-stress [85].

Pro- and anti-inflammatory cytokines help regulate immune responses and prevent excessive inflammation [86]. *IL-1β*, a pro-inflammatory cytokine expressed first after microbial invasion, can stimulate immune responses by enhancing different cellular responses, such as phagocytosis, chemotaxis, and lysozyme synthesis [87]. Its expression is regulated by anti-inflammatory cytokines, including *IL-10* [88]. Regarding gene expression in our study, 100% substitution with BSFM revealed significant upregulation of hepatic *IL-1β* in the pre-challenge period. Similarly, fishmeal replacement with DBSFM improves the relative gene expression of *IL-1β* in European sea bass [62], rainbow trout [89], and groupers [82]. These results stem from the potent antibacterial and immunity-boosting effects of lauric acid found in DBSFM. Contrary to our results, *IL-1β* expression is downregulated in the intestines of Nile tilapia [20] and gilthead seabream [90] fed diets containing defatted BSFM. In the present study, a significant decrease in the expression level of IL*-1β* was observed post-challenge. Similarly, *IL-1β* expression levels in the head kidney at 24-h post-challenge barramundi with *Vibrio harveyi* were downregulated, with no significant effects on the expression level of *IL-1β* in the spleen in groups fed DBSFM supplemented with poultry by-product diets [50].

*TNF-α* is a multifunctional cytokine involved in immune system homeostasis, antimicrobial resistance, apoptosis regulation, cell proliferation, and differentiation [91]. *TNF-α* gene expression in the present study was not affected by dietary DBSFM in the pre-challenge period; however, post-challenge, down-regulation of *TNF-α* expression was observed in all treated groups compared with the control. While other studies showed up-regulation of the mRNA transcripts of *TNF-α* in Koi carp [92], and in marrons (*Cherax cainii*) fed diets supplemented with DBSFLM [93]. Our findings are consistent with our earlier observations regarding MDA levels pre and post-challenge, which suggest no significant accumulation of reactive oxygen species (ROS). These ROS are known to serve as signaling inducers for the pathways that lead to the production of pro-inflammatory cytokines by inhibiting the activation of NF-κB, an important transcription factor that governs cytokine production [94]. Consequently, we did not observe any notable effects prior to the challenge. However, it is worth noting that the opposite trend was seen following the post-challenge phase.

*IL-10* is an essential cytokine for effectively terminating inflammatory responses and restoring homeostasis, marked by the formation of long-lived memory cells to counteract potential threats [95]. *TGF-β1* plays a pivotal role in limiting the inflammatory response and maintaining immune homeostasis by attenuating T-cell activation of T-cells [96]. In the current study, the anti-inflammatory genes *TGF-β1* and *IL-10* mRNA expression levels were upregulated in the DBSFM-post-challenged groups compared to the pre-challenge group. Likewise, *IL-10* gene expression was significantly increased in 24-hour challenged Nile tilapia with bacterial lipopolysaccharide, which was previously fed BSF meal supplemented with chitinase for 53 days [64]. Moreover, barramundi-fed BSFM supplemented with poultry by-products showed upregulation *IL-10* gene in the head kidney 24-h post-challenge with *Vibrio harveyi* [50]. A study by Cardinaletti et al. [89] reported a considerable up-regulation of intestinal *IL-10* in rainbow trout fed on diets supplemented with 25% and 50% full-fatted *H. illucens* prepupae meal. Dietary BSFM up-regulates the relative gene expression of *IL-8* in rainbow trout [97] and the mRNA level of *IL-10* in pearl gentian grouper (*Epinephelus fuscoguttatus* ♀ × *Epinephelus lanceolatus* ♂) [98]. Stenberg et al. [99] demonstrated that 66% and 100% dietary BSFM in the Atlantic salmon improved *IL-10* gene expression. *TGF-β1* is another anti-inflammatory cytokine that plays an important role in preventing excessive inflammation and negatively controls the innate immune system by inhibiting the activity of natural killer cells, macrophages, and neutrophils [100]. The enhanced expression of immune-related genes could be attributed to antimicrobial compounds, such as chitin and chitinase enzymes found in BSFLM, which enhance fish cellular immunity [101, 102]. On the other hand, intestinal expression of pro-inflammatory cytokine genes such as *TNF-a*,* IL-8*,* IFNγ*, and *IL-1b* has been observed in Japanese seabass (*Lateolabrax japonicus*) and salmon [81, 103]. Pro-inflammatory and anti-inflammatory cytokines increased significantly in the control group post-challenge compared with their respective levels before the challenge. This result corresponds to a study by El Aamri et al. [104], where transcription levels of *IL-1β*, *IL-10*, *IL-6*, *TNF-α*, and *IFN-γ* were notably increased in European seabass infected with *S. iniae*.

## Conclusion

This investigation addresses the knowledge gap regarding the effects of **DBSFM**, not only as a replacement for FM but also as a protective agent against bacterial challenges. The findings reveal that substituting FM protein with DBSFM in Nile tilapia improved its immune response with no negative impact on other physiological body functions. Furthermore, the replacement with DBSFM led to improvements in hematological parameters, lysozyme activity, phagocytic activity, and antioxidative indices without causing significant inflammatory changes. Additionally, the use of DBSFM effectively protected the health parameters of Nile tilapia when exposed to bacterial challenges. Future studies are necessary to investigate the ecological implications of using DBLM as a feed source, particularly its sustainability in production and potential impacts on aquatic ecosystems.

## Data Availability

All data supporting the findings of this study are available within the paper.
